# The Impact of Overnight Orthokeratology on Accommodative Response in Myopic Subjects

**DOI:** 10.3390/jcm9113687

**Published:** 2020-11-17

**Authors:** Ana F. Pereira-da-Mota, Jéssica Costa, Ana Amorim-de-Sousa, José M. González-Méijome, António Queirós

**Affiliations:** Clinical and Experimental Optometry Research Lab (CEORLab), Center of Physics, School of Science, University of Minho, Gualtar, 4710-057 Braga, Portugal; ana_filipa96@hotmail.com (A.F.P.-d.-M.); jeka_tatiana@hotmail.com (J.C.); ana.amorim.sousa@gmail.com (A.A.-d.-S.); jgmeijome@fisica.uminho.pt (J.M.G.-M.)

**Keywords:** orthokeratology, accommodation, myopia control, contact lenses

## Abstract

This study aimed to evaluate the effects of two months of orthokeratology (OK) treatment in the accommodative response of young adult myopes. Twenty eyes (21.8 ± 1.8 years) were fitted with the Paragon CRT^®^ 100 LENS to treat myopia between −1.00 and −2.00 D. Low- and high-contrast visual acuity (LCDVA and HCDVA), central objective refraction, light disturbance (LD), and objective accommodative response (using the Grand Seiko WAM-5500 open-field autorefractometer coupled with a Badal system) were measured at baseline (BL) before lens wear and after 1, 15, 30, and 60 nights of OK. Refractive error correction was achieved during the first fifty days of OK lens wear, with minimal changes afterwards. LD analysis showed a transient increase followed by a reduction to baseline levels over the first 30 nights of treatment. The accommodative response was lower than expected for all target vergences in all visits (BL: 0.61 D at 1.00 D to 0.96 D at 5.00 D; 60 N: 0.36 D at 1.00 D to 0.79 D at 5.00 D). On average, the accommodative lag decreases over time with OK lens wear. However, these differences were not statistically significant (*p* > 0.050, repeated-measures ANOVA and Friedman test). This shows that overnight OK treatment does not affect objectively measured the accommodative response of young, low myopic eyes after two months of treatment stabilization.

## 1. Introduction

Orthokeratology (OK) is a clinical contact lens procedure that involves the overnight wear of reverse-geometry rigid gas-permeable corneal contact lenses [1,2]. OK lenses reshape the corneal curvature of the eye during sleep and temporarily correct refractive errors, providing good visual acuity without correction throughout the day

Tackling and avoiding the increased prevalence of myopia has been a constant concern, especially in Asian countries, Europe, and North America. The evidence suggests that besides genetic factors, ethnic differences, less time spent outdoors, increased work, population density, and socioeconomic status impact the prevalence of myopia [3]. Researchers have attempted many methods to reduce the progression of myopia, including pharmaceutical agents, bifocal or multifocal spectacles, soft bifocal/multifocal contact lenses, and OK lenses [4]. OK has been reported to be among the most effective optical strategies [5,6]. This treatment reduces the thickness of the central epithelium and stromal layer and increases the thickness of the midperipheral area of the cornea [7]. The thickening of the midperipheral epithelium and stroma [8] decreases the hyperopic defocus of the peripheral retina in myopic eyes, which might contribute to slow myopia progression [9]. Using OK lenses to slow myopia progression has been studied in recent years, and two meta-analyses were conducted to analyze several controlled studies, indicating a mean reduction in axial elongation of 45% in school-aged children (6–16 years) fitted with OK lenses compared to single-vision corrections over two years of treatment [5,10].

It has been reported that myopic children exhibit larger accommodative lags compared to emmetropic children, even before the onset of myopia [11,12,13], and the resulting hyperopic retinal defocus has been hypothesized as an additional stimulating factor to promote ocular axial elongation [14,15]. To date, an association between abnormal accommodative response and myopia progression and therapeutic interventions to improve the accommodative response either with the induction of negative spherical aberration with contact lenses (CL) or visual therapy failed to slow down myopia progression is unclear [16]. The effect of OK treatment on accommodative response has been the object of several studies and produced controversial results. Computational studies considering actual corneal topographical changes showed that the spherical aberration induced by the treatment might increase the depth of focus in the myopic eye, with a lag of accommodation at near distances being potentially more beneficial in moderate myopes [17,18]. McLeod measured the objective accommodation in children treated with OK and found no changes in the accommodative response measured in low myopes after three months of treatment, although the authors did not specify the baseline measurement conditions [19]. Brand found no changes in accommodative lag but demonstrated a significant improvement in the accommodative facility after three months of lens wear [20]. This study was limited by a small sample size and heterogeneous patients. The author considered the need for more comprehensive research to confirm this result. After evaluating a larger adult population, Felipe-Marquez et al. reported that the accommodative function was not changed by OK treatment for either a short (three months) or long period (three years) [21]. Gifford et al. compared the near-point binocular vision function of young adult myopes wearing OK lenses to single-vision contact lens (SVCL) wearers [22]. The authors reported that the subjects wearing OK lenses displayed more exophoria and lower accommodative lags compared to the SVCL group. However, the clinical methods of all these studies require either the response of the subject, the clinician’s subjective judgment, or both, and using objective methods might bring some clarification to the controversy.

The study aims to investigate the effect of OK treatment in the accommodative response of young adult myopes. To our knowledge, this study is the first evaluating the objective accommodative response using a Badal system to stimulate accommodation coupled with an open-field autorefractometer that measures the corresponding objective refractive change of the eye.

## 2. Material and Methods

### 2.1. Study Design

This prospective, longitudinal study involved participants fitted with OK lenses in both eyes for overnight wear. For the inclusion of subjects, a comprehensive optometric examination was performed and suitability for orthokeratology was assessed. The measurements were taken at baseline (BL) before OK lens wear and repeated after 1 (1 N), 15 (15 N), 30 (30 N), and 60 nights (60 N) of OK lens wear. Baseline measurements were performed through the distance subjective refraction (spherical equivalent refractive error (SER)) with an SVCL measured at the initial examination. The intraocular pressure was verified with a noncontact tonometer before and after treatment [23].

### 2.2. Subjects and Inclusion Criteria

Twenty eyes with myopia were recruited for this study. The inclusion criteria required that the subjects presented an SER distance of −1.00 D to −2.00 D and less than or equal to 1.50 D of astigmatism. No patient had previous or current use of contact lenses and contraindications for OK lens wear. The subjects did not suffer from any eye disease or injury and were not taking any ocular or systemic medication.

Following the recommendations of the Declaration of Helsinki, all subjects received information about the study before they agreed to participate and signed a consent form. The protocol of the study has been reviewed and approved by the Ethics Subcommittee for Life and Health Sciences of the University of Minho (SECVS 066/2015). The study was conducted at the Clinical and Experimental Optometry Research Lab (CEORLab), at University of Minho (Braga, Portugal).

### 2.3. Measurements and Protocol

Central objective refraction: Noncycloplegic central objective refraction was obtained with the open-field Grand Seiko autorefractometer/keratometer WAM-5500 (Grand Seiko Co., Ltd., Hiroshima, Japan) and a mean of 5 measurements is reported.

Visual Acuity: Distance visual acuity was measured with a Logarithmic Visual Acuity Chart “ETDRS—Early Treatment Diabetic Retinopathy Study” (Precision Vision, Woodstock, IL, USA) under high (100%) (high-contrast distance visual acuity (HCDVA)) and low contrast (10%) (low-contrast distance visual acuity (LCDVA)) at 4 m (as recommended by the manufacturer).

Light disturbance (LD): Measurements of light disturbance were performed monocularly with the light disturbance analyzer (LDA) [24] under noncycloplegic conditions. This study evaluated the size (light disturbance index (LDI)) and irregularity (BFC_Irreg_ and BFC_IrregSD_) parameters of LD. The light disturbance index (LDI, %) is the ratio of the area or points missed by the subject and the total area explored. Higher disturbance values (LDI) are interpreted as a lower ability to discriminate small stimuli surrounding the central light source. The irregularity of the disturbance given by BFC irregularity (BFC_Irreg_) (mm) and the SD of BFC_Irreg_ (BFC_IrregSD_) indicates, for each meridian, the difference between the disturbance and the best-of-fit circle and its standard deviation (SD). The higher the value of this parameter, the larger the deviation from a circular shape [25].

**Figure 1:**
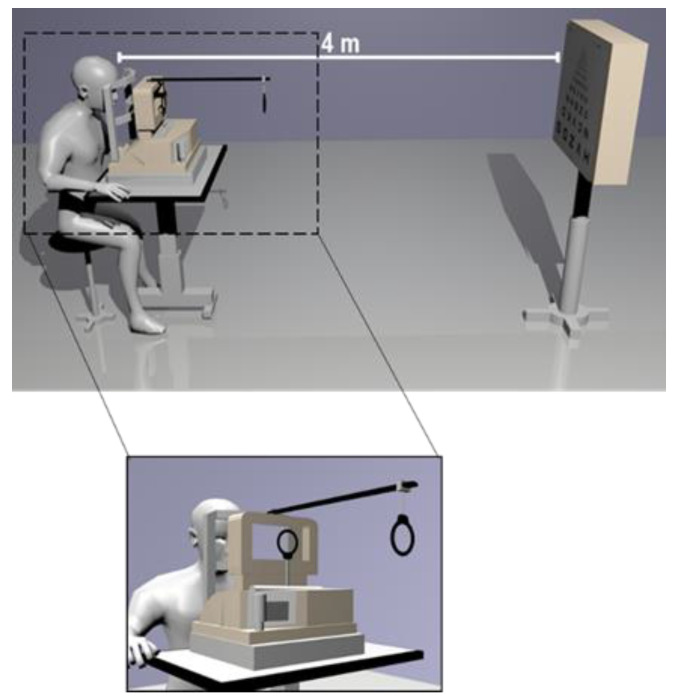
Representation of the monocular Badal system with the ETDRS chart placed at a 4 m distance. A close-up shows the auxiliary and movable lenses in more detail.

Accommodative response: Accommodation measures were obtained with the open-field autorefractometer/keratometer WAM-5500 (Grand Seiko Co., Ltd., Tokyo, Japan). A monocular Badal system was mounted in front of the viewing window, aligned with the eye being evaluated and with a fixed object (ETDRS chart) placed at a 4 m distance (Figure 1). The Badal system consisted of a fixed Badal lens with a focal length of 200 mm. A movable auxiliary lens with a focal length of 150 mm was attached to an automated motor system commanded with a mouse controlled by the patient or the operator through a dedicated software. A custom software (Dynamic Refractive Error Recording (DRER), CEORLab, Braga, Portugal) was coupled with the system to automatically record data from the autorefractometer.

The auxiliary lens was initially moved away from the Badal lens to induce myopia and relax accommodation. The patient was then asked to move the auxiliary lens through a motorized system controlled with a sensitive mouse device until the first position where the ETDRS line of 0.1 logMAR was clear. According to the distance vision over-refraction, the positions of the auxiliary lens to simulate an accommodative demand of 0.00 D (infinity), 1.00 D (1 m), 2.00 D (50 cms), 3.00 D (33 cms), 4.00 D (25 cms), and 5.00 D (20 cms) were calculated. At each position, refraction was measured with the open-field autorefractometer. The difference compared to the distance vision refraction (0.00 D demand) was recorded as the accommodative response. These measurements were performed monocularly with the contralateral eye occluded to avoid convergence misalignments with the Badal and measurement system. Although the monocular accommodative response is not expected to be the same as under real-life binocular conditions, recent results from Altoaimi et al. [26] show that there is no significant difference in the expected response under both conditions either when using monofocal or center-distance multifocal contact lenses. Therefore, it is not expected that the monocular accommodative response in orthokeratology subjects is not representative of the response under binocular conditions when the goal of the present study is to compare the accommodative response of each individual under different conditions. The pupils were under physiological conditions. The evaluation protocol of the objective accommodative response was described in descriptive statistics (mean ± SD) through refraction vector components M, J0, and J45 according to Fourier analysis, as recommended by Thibos [27].

### 2.4. Contact Lens

Subjects were fitted with the Paragon CRT^®^ 100 Lens (sigmoid reverse-geometry rigid gas-permeable lenses—paflufocon D, Dk = 100 barrer— Paragon Vision Sciences, Mesa, AZ, USA) in both eyes for 2 months. OK lenses were fitted by the same practitioner according to the manufacturer’s recommendation, and average lens fitting parameters were as follows: BCR = 8.30 ± 0.25 mm; RZD = 518.75 ± 17.45 μm; LZA = 32.85 ± 0.73°. The flattest SimK (simulated keratometry) values from the corneal topography and the spherical component of the refractive error were used to select the initial diagnostic lens with the sliding tables produced by the manufacturer. Subjects were instructed on the use of OK lenses, including instructions on lens insertion and removal, lens cleaning, and wear regimen. A full optometric eye examination was performed at the follow-up visits, and patients underwent slit-lamp observation for any adverse events and evaluation of OK lens fit. The SVCL used at the baseline visit was a silicone hydrogel contact lens (Biofinity – comfilcon A, CooperVision^®^, Lake Forest, CA, USA).

### 2.5. Statistical Analysis

Statistical analysis was conducted using SPSS v.24.0 (IBM Co., Armonk, NY, USA). The descriptive data are presented as mean ± standard deviation. A total of 20 patients will participate in this two-treatment crossover study. There is an 85% probability that, if the true difference between treatments is 0.250 units, the study will detect a treatment difference at a two-sided 0.05 significance level. This is based on the assumption that the within-patient standard deviation of the response variable is 0.25. The normality of all variables was evaluated using the Shapiro–Wilk test. Considering the nature of data distribution, differences between the different visits were assessed using a repeated-measures Friedman test to compare variables, with post hoc Bonferroni correction for pair comparisons. Differences were considered statistically significant at *p* < 0.05.

## 3. Results

Results from 20 eyes (mean ± SD: 21.8 ± 1.8 years; age range: 20–32 years) are reported. The mean spherical equivalent refractive error of the subjects was −1.50 ± 0.45 D before starting the OK treatment. All participants demonstrated relatively centered bull’s eye OK topography maps.

Table 1 summarizes the central objective refraction and monocular VA distance under high- and low-contrast conditions at BL (through an SVCL with the SER of the subject) and after 1, 15, 30, and 60 nights of OK lens wear with no correction in place. The values after 1 N of OK lens wear increase compared with the BL values in all evaluated parameters (M, J0, J45, HCDVA, and LCDVA) because subjects were corrected on the BL measurement, and the central objective refraction increases as the distance VA decreases. After 1 N until 30 N of OK treatment, there was a hyperopic spherical equivalent shift. Statistically significant differences were found between BL and 1 N, 15 N, 30 N, and 60 N (*p* < 0.001, Friedman test and Bonferroni post hoc test). No significant changes were found in astigmatic components J0 and J45 (*p* > 0.005, Friedman test and Bonferroni post hoc test). The high and low VA distance showed a significant improvement after 1 N until 30 N. The HCDVA were found to have statistically significant differences between 1 N and 15 N, 30 N, and 60 N (*p* < 0.001, Friedman test and Bonferroni post hoc test). The LCDVA showed statistically significant differences between BL and 1 N and between 1 N and 30 N (*p* = 0.004, Friedman test and Bonferroni post hoc test). The measurements of VA taken after 60 N decreased, especially for the LCDVA.

**Figure 2:**
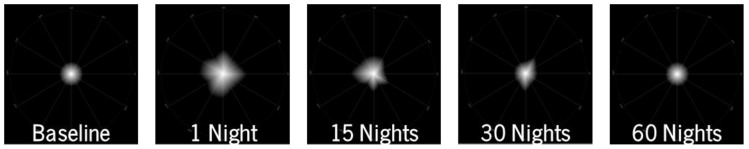
Illustration of the average light disturbance size at BL and after 1, 15, 30, and 60 nights of OK lens wear. The figures represent the mean values of the sample.

Table 2 shows the changes over time of light disturbance parameters: LDI, BFC_Irreg_, and BFC_IrregSD_. Regarding LDI, which represents the halo size, there was an increase after 1 N and 60 N of OK lens wear and a significant decrease at 15 N and 30 N (*p* = 0.007, Friedman test and Bonferroni post hoc test). These results corroborate the values of central objective refraction and VA observed (Table 1). The irregularity parameters did not differ significantly during the treatment. Figure 2 shows an illustration of the mean light disturbance for each visit.

**Figure 3:**
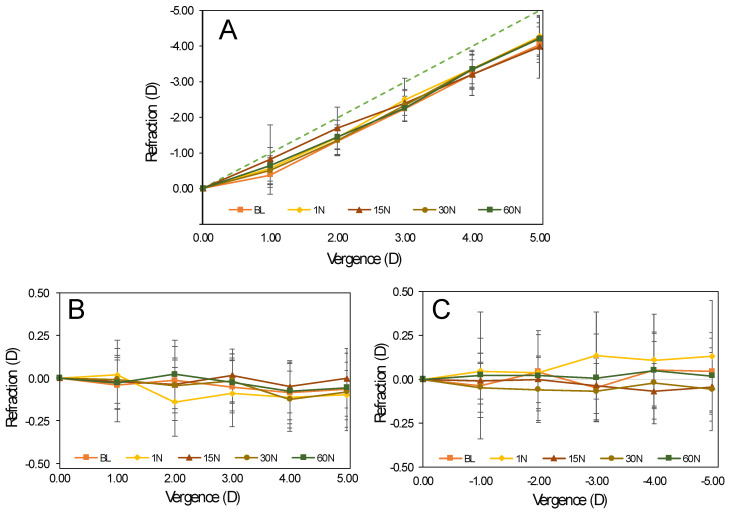
Accommodative response at BL and after 1, 15, 30, and 60 nights of OK lens wear for the spherical equivalent (**A**), J0 component (**B**), and J45 component (**C**). Errors bars represent the standard deviation.

Table 3 presents the accommodative response measured with the open-field autorefractometer and the variation of vector components of astigmatism during accommodation. The values are the difference between the distance vision refraction (0.00 D demand) and the refraction measured for each target vergence. The values presented in Table 3 are shown graphically in Figure 3. The accommodative response was lower than expected for all target vergences in all visits (BL: 0.61 D at 1.00 D to 0.96 D at 5.00 D; 60 N: 0.36 D at 1.00 D to 0.79 D at 5.00 D). On average, the values of accommodative response increase over time with OK lens wear at 1.00 D (0.25 D), 2.00 D (0.12 D), 3.00 D (0.02 D), 4.00 D (0.14 D), and 5.00D (0.17 D). However, these differences were not statistically significant (*p* > 0.050, Friedman test, according to sample distribution). The changes in refractive astigmatism (J0 and J45 components) were lower than 0.20 D. There was only a statistically significant difference for J45 measured at BL–1 N and 1 N–30 N at 3.00 D (*p* = 0.001, Friedman test and Bonferroni post hoc test) and at 1 N–15 N at 4.00 D (*p* = 0.021, Friedman test and Bonferroni post hoc test).

## 4. Discussion

Myopic eyes generally have relative peripheral hyperopia when refracted off-axis along the horizontal meridian, meaning that the refractive error in the periphery of the retina is more hyperopic (light focused behind the retina) compared to central refraction [28]. It has been hypothesized that the presence of relative peripheral hyperopia promotes myopic progression [29]. Multiple studies have reported changes in the shape of the cornea due to OK contact lenses, resulting in a conversion of relative peripheral hyperopic defocus to relative peripheral myopic defocus after orthokeratology [30,31,32,33,34,35]. Queirós et al. found that the amount of peripheral myopia induced is linearly related to the amount of myopia to be corrected [30]. Therefore, treating higher amounts of myopia resulted in broader myopic shifts in peripheral refractive error [34].

The present study characterized the visual performance and accommodative response changes after successful two months’ OK lens wear in young adult myopes. After 1 N of OK lens wear, an increase in M, J0, and J45 of central objective refraction and a decrease in visual performance occurred (Table 1). It happens because the BL measurements were made through an SVCL with the subject’s spherical equivalent refraction, and the other measurements were made with no correction in place. More significant central refractive changes occurred after the first night and during the first fifteen days (0.50 D) of treatment. After that, the differences were minimal (0.07 D). Nichols et al. conducted a prospective study of refractive error changes in patients undergoing overnight OK and found that most of the changes in refractive variables occurred in the first seven nights of contact lens wear [36]. After the seventh day, there was no significant reduction in myopia. Other studies reported similar results where most of the refractive error reduction occurred within the first week of OK [37,38]. At the 60 N visit, a slight increase in central objective refraction occurred, possibly because the subjects might not have slept every night with the OK lens. There were no significant differences in astigmatism during the OK treatment (Table 1). Other studies have reported that OK generally does not induce astigmatism in patients with spherical refractive errors, which is in agreement with our study [39,40].

After one night of OK lens wear, the uncorrected VA reduced compared to the BL measurements made with the SVCL (Table 1). This reduction is associated with the fact that, in just one night, the amount of myopia is not fully corrected. A meta-analysis by Li et al. found that a reduction in corneal thickness occurs within the first week of OK [41]. As expected, OK leads to a significant improvement in uncorrected HCDVA due to the elimination of uncorrected myopia, demonstrating that increased high-order aberrations do not have a clinically meaningful impact on HCDVA [42,43,44]. After 30 nights of OK, we observed a reduction of less than 0.02 logMAR (one letter). Other researchers have reported similar results [36,37]. Uncorrected LCDVA was reduced by 0.07 logMAR (approximately three letters) compared to SVCL correction, which might be associated with increases in spherical aberration [42].

LD analysis showed a transient increase followed by a reduction to baseline levels over the first 30 nights of treatment (Table 2). Changes observed from 30 nights to 60 nights were not significant. These results are in agreement with previous works by Santolaria et al. [45,46].

To our knowledge, the present study is the first evaluating the objective accommodative response to different vergence targets with a double-lens Badal system. This method allows changing the vergence without affecting the size and luminance of the target. In this sense, pupil size changes are only due to the increase in accommodative demand. The accommodative response slightly improves during OK lens wear, showing lower accommodative lag after 60 nights, but these changes were not statistically significant (Table 3 and Figure 2). Several studies have examined accommodative changes with OK. Felipe-Marquez et al. and Kang et al. did not find significant changes in accommodative function in young myopic adults after three months and 28 nights of OK, respectively [21,47]. However, Gifford et al. found a lower accommodative lag in OK subjects in a retrospective study [22]. The same author, in a 12-month prospective study, reported improved accommodative responses with OK. The accommodative lag decreased after one month of OK in children and after 12 months in young adults [48]. The strongest evidence of changes in the accommodative response with OK is present in a study conducted by Han et al. [49]. This was a randomized study with 240 myopic children assigned to wear SV spectacles (*n* = 90), orthokeratology (*n* = 90), or multifocal spectacles (*n* = 60). In children wearing OK, accommodative lag was significantly lower after one year compared to BL measurements and the SV spectacles group. This increase in accommodation after a long-term OK could be an adaptation to counteract the increase in positive spherical aberration caused by OK [50]. The results of the present study do not support a decrease in accommodation due to the extended depth of field induced by the positive spherical aberration induced by the OK treatment [18]. Instead, it seems that an increase in accommodation response might occur for intermediate vergences in this sample of low myopes after OK treatment, though this effect is not statistically significant (Table 3 and Figure 3A).

The authors recognize some limitations in this study. These results are only applicable to young- adult subjects and under monocular conditions. The measurements were made monocularly, and the accommodative response obtained might not be representative of that shown under binocular conditions. However, for this study, this limitation should have no impact when comparing the response in different times of the treatment, and as mentioned in the Section 2, it is not expected that under the conditions of this experiment, the response would be significantly different. Despite the results presented by Felipe-Marquez et al. [51], who showed that OK does not significantly change the binocular function for either short- or long-term periods, in a further investigation, it would be interesting to evaluate the accommodative response with the same method and with a larger sample size, subjects with different degrees of myopia, and a long-term group. Batres et al. recently evaluated the changes in accommodative LAG using Nott retinoscopy and concluded that there was a reduction of about 0.25 D in accommodative lag from baseline [52]. Although this is an objective test, the clinician’s judgment plays a critical role in the results. Furthermore, the retinoscopic reflex in orthokeratology patients is significantly distorted, which might also affect the judgment ability to find neutralization. In our study, the same objective system, analyzing the same pupil area between exams, was applied. Our understanding is that the difference in methodologies is the reason for the controversial results between studies, which is necessary to evaluate the accommodative response with objective methods without subject or observer intervention.

## 5. Conclusions

The results of our study suggest that OK lens wear slightly improves the accommodative response. However, these changes are not clinically significant. Therefore, we can conclude that, in general terms, OK does not influence the accommodative response over short periods of treatment in young adult low myopes.

## Figures and Tables

**Table 1:** Objective spherical equivalent central refraction or M (D; mean ± SD), astigmatic power vectors at 0° and 45° and distance VA under high-contrast distance visual acuity (HCDVA) and low-contrast distance visual acuity (LCDVA) (LogMAR units; mean ± SD) at baseline (BL) and after 1, 15, 30, and 60 nights of orthokeratology (OK) lens wear.

	M (D)	J0 (D)	J45 (D)	HCDVA	LCDVA
BL	−0.33 ± 0.68	0.05 ± 0.22	−0.02 ± 0.21	−0.09 ± 0.08	0.08 ± 0.08
1 N	−0.52 ± 0.38	0.07 ± 0.18	−0.03 ± 0.23	0.02 ± 0.11	0.25 ± 0.13
15 N	0.02 ± 0.40	0.01 ± 0.15	−0.06 ± 0.26	−0.09 ± 0.10	0.11 ± 0.12
30 N	0.00 ± 0.42	−0.01 ± 0.19	−0.07 ± 0.21	−0.12 ± 0.08	0.09 ± 0.11
60 N	−0.05 ± 0.36	−0.03 ± 0.15	0.01 ± 0.24	−0.11 ± 0.11	0.15 ± 0.13
*p*-values	<0.001+	0.390+	0.359+	<0.001+	0.004+
*Post hoc* test	1 N–BL;1 N–15 N; 1 N–30 N; 1 N–60 N	x	x	1 N–15 N; 1 N–30 N; 1 N–60 N	BL-1 N; 1 N–30 N

+—Friedman test and Bonferroni post hoc test. Statistically significant differences among the visits are bolded. Example of pair-by-pair comparison. 1 N–15 N—statistically significant differences between 1 and 15 nights. x—non-statistically significant differences with a pair-by-pair comparison.

**Table 2:** Light disturbance index (LDI) (%; mean ± SD), irregularity of the disturbance or BFCIrreg and the SD of BFCIrreg (mm; mean ± SD) at BL and after 1, 15, 30, and 60 nights of OK lens wear.

	LDI (%)	BFC_Irreg_ (mm)	BFC_IrregSD_ (mm)
BL	10.50 ± 5.59	0.35 ± 0.25	3.24 ± 1.16
1 N	11.31 ± 5.96	0.46 ± 0.33	3.27 ± 1.16
15 N	9.57 ± 5.90	0.60 ± 0.82	3.52 ± 1.87
30 N	9.79 ± 8.18	0.71 ± 1.37	2.86 ± 2.14
60 N	10.51 ± 7.98	0.50 ± 0.68	3.23 ± 2.28
*p*-values	0.007+	0.821+	0.321+
*Post hoc* test	1 N–15 N; 1 N–30 N	x	x

(+) Friedman test and Bonferroni post hoc test. Statistically significant differences among the visits highlighted in bold. Example of pair-by-pair comparison. 1 N–15 N—statistically significant differences between 1 and 15 nights. x—non-statistically significant differences with a pair-by-pair comparison.

**Table 3:** Mean ± standard deviation of the accommodative response (M) and the variation of astigmatic components (J0 and J45) for each target vergence.

		BL	1 N	15 N	30 N	60 N	*p*-values	*Post hoc* test
	1.00 D	−0.39 ± 0.36	−0.57 ± 0.37	−0.82 ± 1.00	−0.50 ± 0.37	−0.64 ± 0.53	0.184+	x
	2.00 D	−1.34 ± 0.39	−1.44 ± 0.51	−1.70 ± 0.61	−1.36 ± 0.44	−1.46 ± 0.35	0.047+	x
M	3.00 D	−2.24 ± 0.36	−2.50 ± 0.62	−2.41 ± 0.36	−2.34 ± 0.47	−2.26 ± 0.36	0.351+	x
	4.00 D	−3.21 ± 0.43	−3.35 ± 0.51	−3.20 ± 0.59	−3.35 ± 0.55	−3.35 ± 0.42	0.721+	x
	5.00 D	−4.04 ± 0.51	−4.28 ± 0.58	−3.98 ± 0.91	−4.23 ± 0.61	−4.21 ±0.46	0.380+	x
	1.00 D	−0.04 ± 0.22	0.02 ± 0.21	−0.02 ± 0.13	−0.01 ± 0.15	−0.03 ± 0.15	0.965+	x
	2.00 D	−0.01 ± 0.24	−0.14 ± 0.21	−0.04 ± 0.14	−0.04 ± 0.17	0.02 ± 0.17	0.115+	x
J0	3.00D	−0.05 ± 0.16	−0.09 ± 0.20	0.02 ± 0.16	−0.02 ± 0.14	−0.03 ± 0.17	0.278+	x
	4.00 D	−0.09 ± 0.18	−0.11 ± 0.20	−0.05 ± 0.16	−0.12 ± 0.17	−0.08 ± 0.17	0.688+	x
	5.00 D	−0.07 ± 0.16	−0.10 ± 0.15	0.00 ± 0.18	−0.08 ± 0.23	−0.06 ± 0.24	0.631+	x
	1.00 D	−0.06 ± 0.24	0.05 ± 0.19	-0.01 ± 0.11	−0.05 ± 0.14	0.02 ± 0.37	0.103+	x
	2.00 D	0.04 ± 0.22	0.04 ± 0.22	0.00 ± 0.14	−0.06 ± 0.19	0.02 ± 0.26	0.366+	x
J45	3.00 D	−0.05 ± 0.19	0.14 ± 0.26	−0.04 ± 0.16	−0.07 ± 0.16	0.01 ± 0.26	0.001+	BL-1 N; 1 N–30 N
	4.00 D	0.05 ± 0.22	0.11 ± 0.27	−0.07 ± 0.16	−0.02 ± 0.24	0.05 ± 0.22	0.021+	1 N–15 N
	5.00 D	0.04 ± 0.23	0.13 ± 0.33	−0.07 ± 0.16	−0.06 ± 0.24	0.02 ± 0.22	0.272+	x

(+) Friedman test and Bonferroni post hoc test. Statistically significant differences among the visits highlighted in bold. Example of pair-by-pair comparison. 1 N–15 N—statistically significant differences between 1 and 15 nights. x—non-statistically significant differences with a pair-by-pair comparison.
